# Dynamic Analysis of an Underwater Cable-Driven Manipulator with a Fluid-Power Buoyancy Regulation System

**DOI:** 10.3390/mi11121042

**Published:** 2020-11-26

**Authors:** Tong Wang, Zihao You, Wei Song, Shiqiang Zhu

**Affiliations:** 1 Ocean College, Zhejiang University, Zhoushan 316021, China; 3160100561@zju.edu.cn (T.W.); youzh8@163.com (Z.Y.); sqzhu@sfp.zju.edu.cn (S.Z.); 2 Zhejiang Lab, Hangzhou 311100, China; 3 Institute of Robotics, Zhejiang University, Yuyao 315400, China; 4 The Engineering Research Center of Oceanic Sensing Technology and Equipment, Ministry of Education, Zhoushan 316021, China

**Keywords:** underwater manipulator, cable-driven manipulator, buoyancy regulation, dynamic modeling

## Abstract

This article presents an underwater cable-driven manipulator (UCDM) with a buoyancy regulation system (BRS), which is controlled by a fluid-power system. The manipulator consists of five sections, and each section is embedded with a buoyancy adjustment unit. By regulating buoyancy at each section, the static and dynamic states of the manipulator will be changed, promising a new operating mode of an underwater manipulator driven by buoyancy. In this article, a dynamic model of the manipulator is established by the Newton-Euler equation, considering cable tension, inter-joint force, buoyancy, water resistance and other variables. With a numerical method, the dynamic model is solved and the values of cable tension are obtained, which are used to evaluate the buoyancy-driven operating mode of underwater manipulator. This research will be useful for manipulator operating in fluid environments, such as underwater manipulator in the ocean, micro-manipulator in a blood vessel, and so on.

## 1. Introduction

Underwater manipulators are widely applied in the exploitation of ocean resources, which can assist people in multiple tasks such as collecting marine samples [1], gripping underwater objects [2], operating and maintaining underwater equipment of oil and gas [3]. They are now indispensable engineering equipment for marine development [4,5,6,7,8].

Traditional underwater manipulators mainly adopt a rigid structure with a large body mass and moment of inertia. Therefore, they are not suitable for operations in narrow and complicated environments such as underwater pipelines and sunken ships [9,10]. In comparison, the cable-driven manipulator is widely used in fields like nuclear power [11,12], medical [13,14] and aviation [15,16], which are above the water, due to its high degree of freedom (DOF) and large workspace [13,14,17,18,19,20]. If the cable-driven manipulator can be applied in underwater scenes, it will have a significant potential in marine exploration and resource development [2,10], which is also meaningful for the development of manipulators operating in fluid environments like micro-manipulators used in medical fields [21,22,23,24].

Increasing the number of joints and the length of the arm can increase the flexibility of the cable-driven manipulator and expand its range of operation. However, this also increases the self-weight of the manipulator, resulting in additional power consumption of motors to balance the impact of the self-weight. At present, researchers have carried out some research on this issue. Thrusters, such as water jets [25] and propellers [26], are installed on the manipulator to compensate the manipulator’s self-weight. In addition, Masashi Takeichi’s team developed the Giacometti series of manipulators, which balance the weight of the manipulator through buoyancy generated by helium gas [27]. Since the buoyancy-driven scheme neither needs to consume energy continuously, nor to install thrusters on the manipulator, the scheme proposed by reference [27] has more advantages than that of references [25,26]. Therefore, we design a fluid-power buoyancy regulation system for an underwater cable-driven manipulator (UCDM). It is expected that the buoyancy regulation system (BRS) could weaken the influence of the UCDM’s self-weight, thereby helping to improve the operating performance of the UCDM.

In recent years, researches on cable-driven manipulators are mainly aimed at on-land operations, focusing on the motion control [28,29,30], kinematic [31,32] and dynamic [33,34] modeling. These studies rarely involve underwater applications [10], and have not yet combined buoyancy driving with UCDM. For the underwater operation environment, hydrodynamic factors such as buoyancy and water resistance make the dynamic model of the UCDM more complicated. Guohua Xu et al. studied the influence of hydrodynamic factors on the dynamics of an underwater manipulator [35]. They found that the buoyancy has the greatest impact on underwater manipulators, which is the same order of magnitude as the gravity of the manipulator. Therefore, for the buoyancy-driven scheme, it is of great significance to establish the dynamic model of the UCDM and to explore the effect of buoyancy on the manipulator [36,37].

In the dynamic model, the cable tension directly influents the motion state of the UCDM, and reflects BRS’s effect on the UCDM. Therefore, our research focuses on BRS’s effect on the cable tension during the action of the UCDM in underwater operations.

In this article, an underwater cable-driven manipulator with a fluid-power buoyancy regulation system is firstly proposed. The UCDM is composed of five arm sections with embedded BRS, which can compensate gravity by regulating buoyancy in each section. Secondly, based on the mechanical structure and working principle of the UCDM, a dynamic model is established using Newton–Euler method, including cable tension, inter-joint force, buoyancy, water resistance and other variables. We solve the dynamic model through numerical calculation and obtain the time-varying value of each variable. Then we analyze BRS’s effect on the cable tension of the UCDM. Finally, a conclusion is drawn providing a theoretical basis for a future buoyancy-driven scheme.

## 2. Mechanical Structure and Working Principle

### 2.1. Overall Structure of the Manipulator

**Figure 1:**
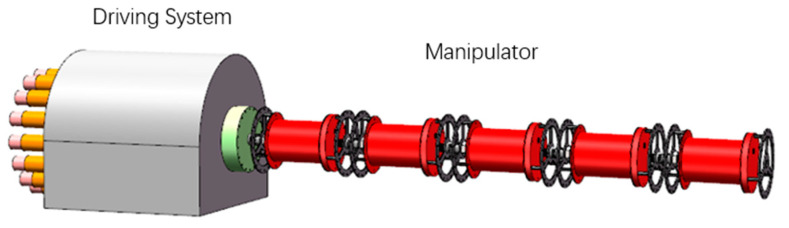
Mechanical structure of the manipulator.

The overall mechanical structure of the UCDM proposed in this article is shown in Figure 1. It consists of a driving system and a cable-driven manipulator. The driving system includes cables and motors mounted on the base. The manipulator is composed of five arm sections with embedded BRS. Adjacent sections are coupled by cross universal joints. According to task requirements, the manipulator can expand the number of sections to improve its adaptability in operations. Considering the need of self-weight reduction and rust prevention, 6061 aluminum alloy is selected as the main material of the UCDM. The specifications of the manipulator are shown in Table 1.

### 2.2. Structure of a Section

**Figure 2:**
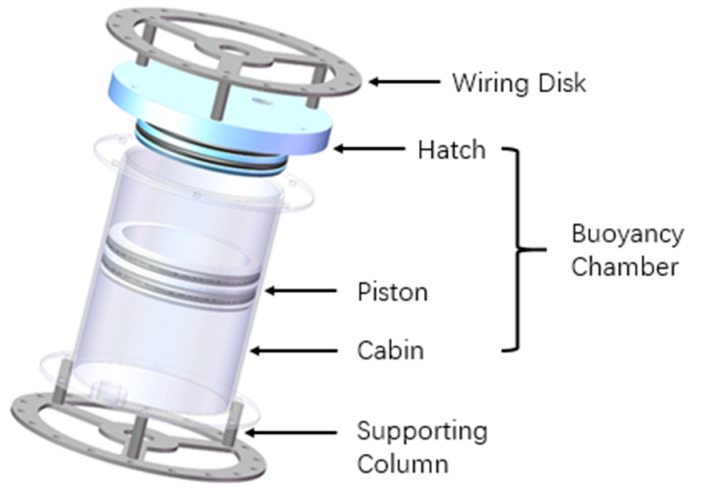
Structure of a section.

As shown in Figure 2, an arm section consists of two wiring disks, six supporting columns, and a buoyancy chamber. The buoyancy chamber mainly consists of a cabin, a hatch, a piston, two nozzles and several sealing rings. The piston separates liquid and gas, so the buoyancy can be adjusted by a fluid power system, which changes the proportion of liquid and gas in the chamber. Each two adjacent sections are connected by a universal joint fixed on wiring disks by nuts, so that each joint has a rotation range of ±30° in two degrees of freedom (DOFs) of movement.

### 2.3. Working Principle of Buoyancy Regulation System (BRS)

**Figure 3:**
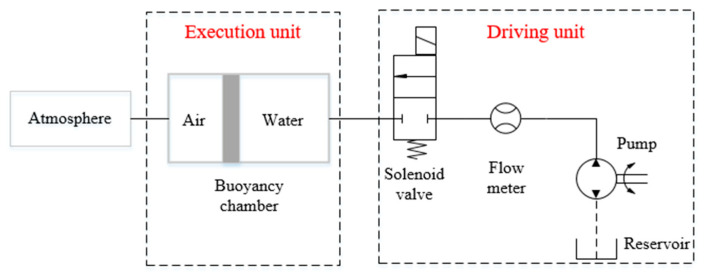
Working principle of buoyancy regulation system (BRS).

The BRS of each section is composed of micro pump, flow meter, solenoid valve and connecting pipelines. Its working principle is shown in Figure 3. The liquid medium in the buoyancy chamber is water, and the gas medium is air. The buoyancy of each section is adjusted by changing the volume of water in the chamber by the pump control system. The driving unit of the BRS is installed on the base, connected to the execution unit through pipelines. This avoids the waterproof problem of the electrical system and simplifies the structure’s design.

## 3. Modeling

### 3.1. Kinematics

**Figure 4:**
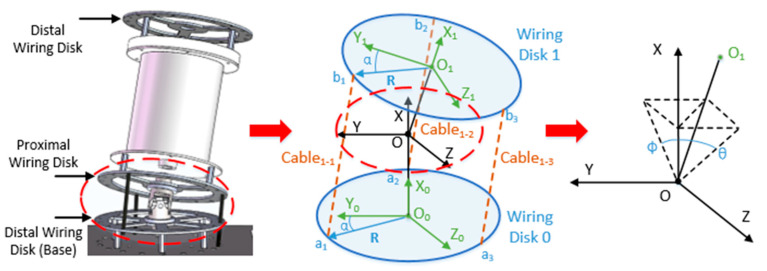
Joint model of the manipulator.

We create a geometric model of a joint, as shown in Figure 4. We create frame {O}, frame {O_2k−2_}, and frame {O_2k−1_} at the center of rotation of the universal joint, the center of wiring disk 2k − 2, and the center of wiring disk 2k − 1, respectively (k is the section number from the base, k = 1, 2, 3, …, n). R represents the distance from the wiring hole to the center of the wiring disk, and α represents the angle of **R** relative to the Y axis of the disk, which is called the cable deflection angle (bold indicates vector, the same below). The two rotation angles of frame {O_2k−1_} relative to frame {O_2k−2_} are θ_i_ (refer to the Z axis) and φ_i_ (refer to the Y axis). In addition, let OO_2k−2_ = OO_2k−1_ = D, O_2k−1_O_2k_ = H.

Assuming that the UCDM has n sections in total, α_i,j_ represents the cable deflection angle of the j-*th* cable that controls the i-*th* joint (i = 1, 2, 3,..., n; j = 1, 2, 3). Each section is controlled by 3 cables, and their cable deflection angles differ by 2π/3. Assuming that each disk has s wiring holes evenly distributed in the circumferential direction, let $\alpha_{1,1}=0$, then we have:

(1) $$\alpha_{i,j}=(i-1)\times \frac{2\pi }{s}+(j-1)\times \frac{2\pi }{3}.$$

From (1), the coordinate of a_1_ in frame {O_2k−2_} and the coordinate of b_1_ in frame {O_2k−1_} are both (0, R cosα_i,j,_ R sinα_i,j_). Then we only require the coordinate of b_1_ in frame {O_2k−2_} to derive the distance between a_1_ and b_1_. This distance represents the length of the j-*th* cable controlling the i-*th* joint between the k-*th* and (k − 1)-*th* section, denoted as l_i,j,k_ (i ≥ k). Frame {O_2k−1_} can be derived from frame {O_2k−2_} through the transformation matrix Tr:

(2) $$\mathrm{Tr}=\mathrm{Trans}(D,0,0)\mathrm{RotZ}(\theta_{i})\mathrm{RotY}(\varphi_{i})\mathrm{Trans}(D,0,0),$$

where Trans() represents the translation function, RotZ() and RotY() represent the rotation function refer to Z-axis and Y-axis. Let the coordinate of b_1_ in frame {O_2k−1_} and frame {O_2k−2_} be p_i,j,k_ and Tr × p_i,j,k_, respectively, we have:

(3) $$l_{i,j,k}=\left\|a_{1}b_{1}\right\|=\left\|\mathrm{Tr}\times p_{i,j,k}-p_{i,j,k}\right\|.$$

Then, the length of the j-*th* cable controlling the i-*th* joint can be derived as follows:

(4) $$L_{i,j}=-H+\sum_{k=1}^{i}\left(l_{i,j,k}+H\right).$$

Furthermore, the transformation matrix from the base frame {O_0_} to the frame at the end of the k-*th* section (that is, frame {O_2k_}) can be derived as follows:

(5) T0kr=∏l=1k(T2l−22l−1r×Trans(D,0,0)).

where T2l−22l−1r represents the transformation matrix from frame {O_2l−2_} to frame {O_2l−1_}.

For any wiring hole p_i,j,k_ on wiring disk 2k, its coordinate at the same position in the base frame {O_0_} is p_i,j,0_. From (5), the coordinates of p_i,j,k_ in the base frame {O_0_} can be derived as follows:

(6) pi,j,k=T0kr×pi,j,0.

### 3.2. Dynamics

The basic goal of the dynamic analysis in this article is to solve all the cable tensions under the condition of known pose and motion state of the manipulator. The mechanical analysis is based on the following assumptions:

1. The links and the joints are assumed as rigid body;
2. The deformation and the mass of the cables are neglected;
3. The tension is equal at every point on the same cable. The cable only transmits tension but not pressure, that is: we always have $T_{k,j}\geq 0$.

**Figure 5:**
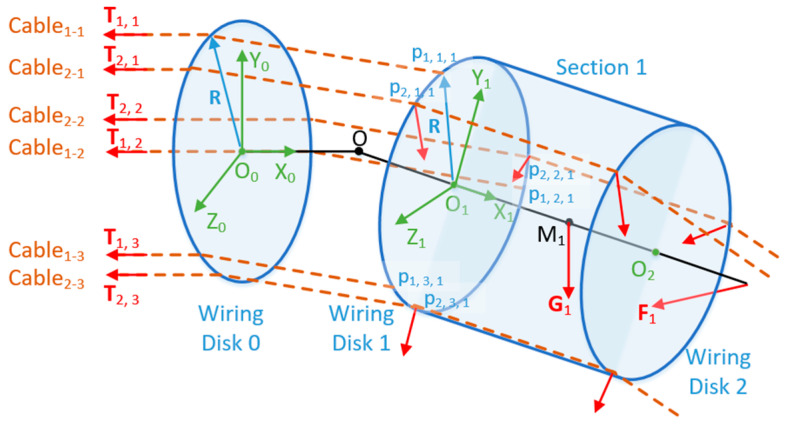
Mechanical analysis of a section.

Assuming that the wiring disk 2k − 2 is a fixed wiring disk (as shown in Figure 5, taking the base disk as an example, k = 1), its coordinate system is the ground coordinate system. Define UCDM’s end close to the base as the proximal end, and the end away from the base as the distal end. Then in Figure 5, the wiring disk 2k − 1 is the proximal wiring disk of section k, and the wiring disk 2k is the distal wiring disk of section k. After analysis, the forces on section k include the following:

1. The inertial force $-m_{k}*a_{k-1}$ and moment of inertia $-J_{k}*A_{k-1}$ generated by the movement of the first (k − 1)-*th* sections from the base (it should be noted that a,A,v,ω, g, etc. mentioned in this section are all vectors in frame {O_2k−1_}. If there is a vector in the ground coordinate system, it needs to be transformed to frame {O_2k−1_} using the transformation matrix);
2. Gravity $G_{k}$;
3. Buoyancy $B_{k}$;
4. The supporting force $F_{k-1}$ and torque $M_{k-1}$ from the former section;
5. The reaction torque $-M_{k}$ and reaction force $-F_{k}$ of the supporting force from the latter section;
6. Pressures generated by the cables (due to bending) passing through the proximal wiring disk and the distal wiring disk, which are $N_{i,\;j,\;k,\;\mathrm{pro}}$ and $N_{i,\;j,\;k,\;\mathrm{dis}}$;
7. The friction force generated by the cable passing through proximal and distal wiring disks, which are $f_{i,\;j,\;k,\;\mathrm{pro}}$ and $f_{i,\;j,\;k,\;\mathrm{dis}}$;
8. The resultant force $T_{k,\;j}$ (approximately equal to the pulling force, explained later) of the pulling force, frictional force and other forces caused by the cable connected to the proximal wiring disk;
9. The water resistance $Fd_{k}$.

To get each force and torque, let the moment of inertia of the section around O be J_k_, and the angular acceleration be **A**_k_. Then we have:

(7) $$J_{k}*A_{k}=\sum \tau_{k},$$

where:

(8) $$J_{k}=J_{0}+J_{water},$$

where J_0_ is the moment of inertia of the mechanical structure, and J_water_ is the moment of inertia of the water in the buoyancy chamber of this section.

$\sum \tau_{k}$ is the sum of the moments on point O, which are produced by forces and torques listed in 1–9 above, including:

1. Moment of inertia:
  (9) $$\tau_{I,k}=-J_{k}*A_{k-1};$$
2. Moment of gravity:
  (10) $$\tau_{G,k}=G_{k}\times OM_{k},$$
  where:
  (11) $$G_{k}=g*m_{k},$$
  (12) $$m_{k}=m_{0}+m_{\mathrm{water}},$$
  where m_0_ is the mass of the mechanical structure, and m_water_ is the mass of the water in the buoyancy chamber of this section. $OM_{k}$ is the force arm of gravity, and the coordinates of M_k_ can be obtained by calculating the position of the centroid;
3. Moment of buoyancy:
  (13) $$\tau_{B,k}=B_{k}\times (D+\frac{H}{2}),$$
  and since the structure of each section is the same, its buoyancy is a constant (the direction will change when converted to frame {O_2k−1_}). The buoyancy acts on the midpoint of O_1_O_2_, so its force arm is $D+\frac{H}{2}$;
4. The torque $M_{k-1}$ from the former section ($F_{k-1}$ does not produce a torque to O);
5. The reaction torque $-M_{k}$ and the torque of reaction force $-F_{k}$:
  (14) $$\tau_{F,k}=-F_{k}\times (2*D+H),$$
  where the force arm is 2∗D+H;
6. Since the mass of each point on the cable is negligible, the resultant force of friction and pressure on the point is equal to the resultant force of the cable tension on both sides of the point. We call this resultant force the deformation force. For cable L_i,j_, the deformation forces on the proximal wiring disk of the k-*th* section are:
  (15) $$NN_{i,j,k,pro}=T_{i,j}*(H-l_{i,j,k}),$$
  their force arms are $D+R_{i,j}$, and their torques are:
  (16) $$\tau_{NN,k,pro}=\sum_{i=k+1}^{n}\sum_{j=1}^{3}NN_{i,j,k,pro}\times (D+R_{i,j}).$$

The deformation forces on the distal wiring disk are:

(17) $$NN_{i,j,k,dis}=T_{i,j}*(l_{i,j,k}-H),$$

their force arms are $H+D+R_{i,j}$, and their torques are:

(18) $$\tau_{NN,k,dis}=\sum_{i=k+1}^{n}\sum_{j=1}^{3}NN_{i,j,k,dis}\times (H+D+R_{i,j});$$

7. Similar to 6., the resultant force of one cable controlling this section can be approximated as the cable tension at this point. Then the torques of cable tensions are:
  (19) $$\tau_{T,k}=\sum_{j=1}^{3}T_{k,j}\times (D+R_{i,j}),$$
  where the cable tension $T_{k,j}$’s direction is $-l_{i,j,k}$, and its magnitude $T_{k,j}$ is the unknown quantity to be obtained. The force arms of the tensions are $D+R_{i,j}$;
8. Moment of water resistance:
  (20) $$\tau_{Fd,k}=Fd_{k}\times (D+\frac{H}{2}).$$

The magnitude of water resistance Fd can be calculated by (21) [38].

(21) Fd=Cd(Re)∗v2∗S∗ρ,

where,

Cd: the drag coefficient;
Re: the Reynolds number that reflect the flow characteristics;
v: the relative velocity of spherical underwater robot to the fluid;
S: the cross-sectional area;
ρ: the density of the fluid.

The direction of water resistance is opposite the direction of section movement. It acts approximately on the midpoint of O_1_O_2_, so its force arm is $D+\frac{H}{2}$.

This is a redundancy problem using three cables to determine a plane. But under the constraints of assumption 3., Equation (7) can obtain a unique solution. Substitute the obtained $T_{k,\;j}$ into the dynamic model established above. Let the translational acceleration of this section be $a_{k}$, we have:

(22) $$M_{k}*a_{k}=\sum force_{k},$$

then the last unknown force $F_{k-1}$ is obtained.

So far, the dynamic state of the section has been uniquely determined.

## 4. Solution and Discussion

### 4.1. Solution

**Figure 6:**
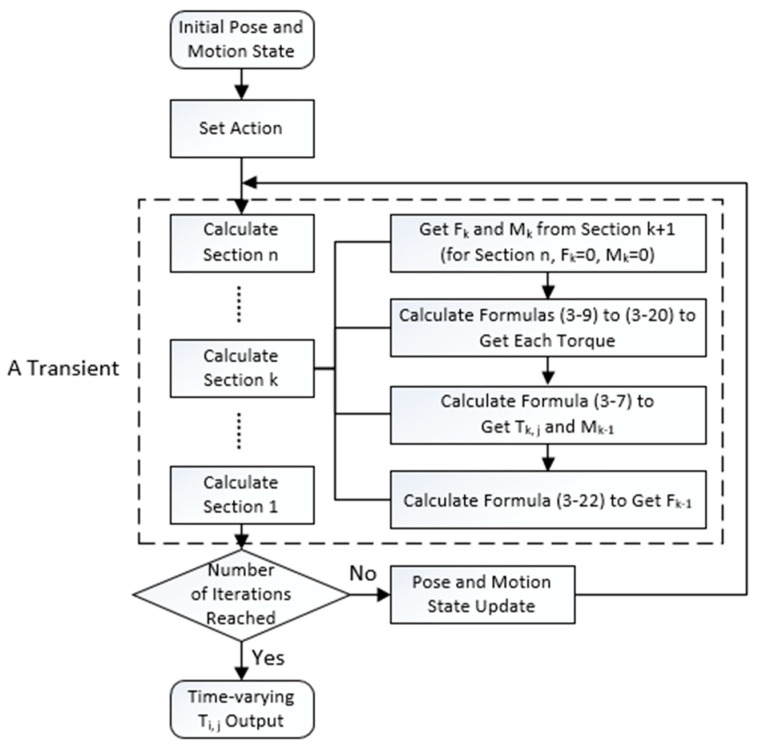
Steps for numerical solution of the dynamic model.

In this section, we numerically solve the dynamic model of the UCDM prototype, as shown in Figure 6. We discretize the motion into multiple transients, and each transient solves the current parameters based on the parameters preset and obtained from the previous transient (the preset parameters mainly include angular acceleration and piston moving speed, and the solved parameters in each transient mainly include angular velocity, pose, and tension). For each transient, the program calculates section by section from the distal end to the proximal end. In the calculation process, **F**_k−1_ and **M**_k−1_ of each section are used as −**F**_k_ and −**M**_k_ of the former section. Hence, we can simulate the motion of UCDM and obtain the time histories of all cable tensions during the motion.

**Figure 7:**
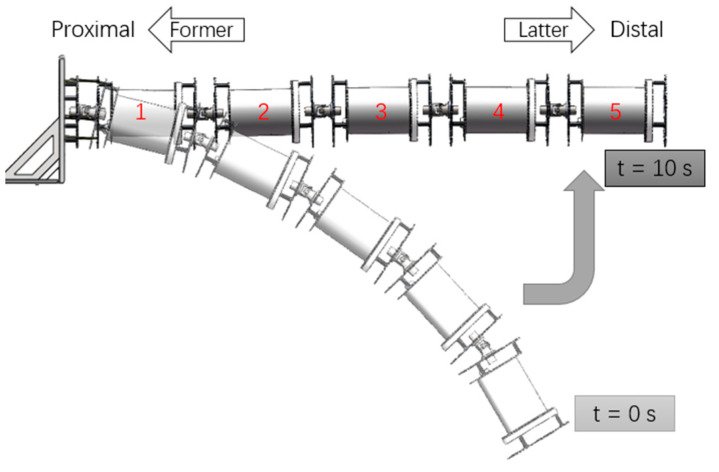
Movement of the manipulator.

Since the buoyancy only acts in the vertical direction, we solve the movement of the manipulator in the vertical plane numerically. The angles of the starting posture and the end posture are assumed as follows. The movement represents the action of the manipulator to lift an object from a low position, as shown in Figure 7.

$$\mathrm{Starting}\;\mathrm{posture}:{\;\theta }_{i}=-0.45;{\;\varphi }_{i}=0;\;\mathrm{end}\;\mathrm{posture}:{\;\theta }_{i}=0;{\;\varphi }_{i}=0.$$

**Figure 8:**
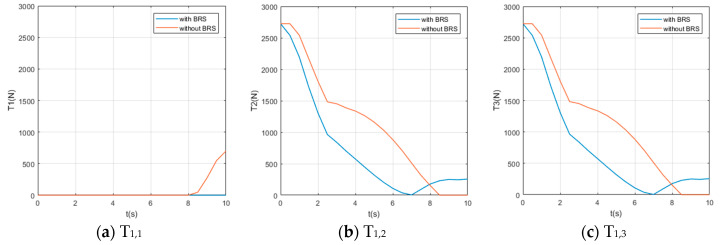
The time histories of the tensions on cables controlling Section 1.

**Figure 9:**
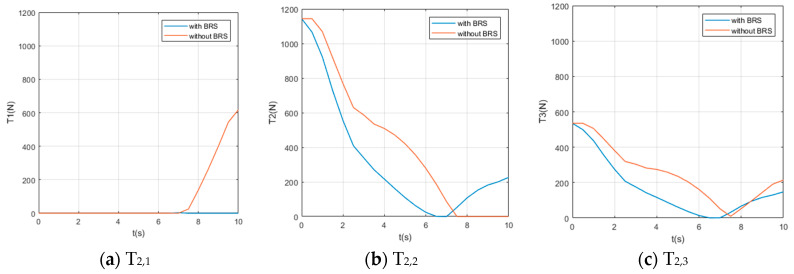
The time histories of the tensions on cables controlling Section 2.

**Figure 10:**
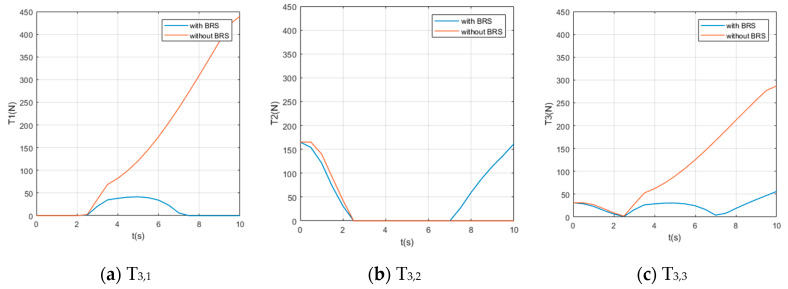
The time histories of the tensions on cables controlling Section 3.

**Figure 11:**
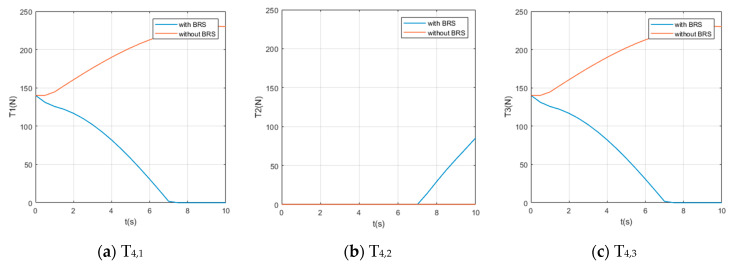
The time histories of the tensions on cables controlling Section 4.

**Figure 12:**
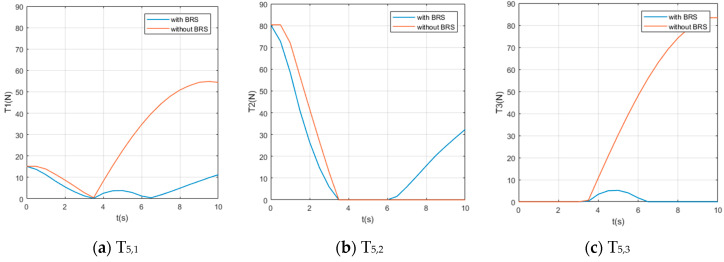
The time histories of the tensions on cables controlling Section 5.

We also assume the movement starts and ends with 0 velocity and a certain acceleration (in order to start or stop the manipulator’s movement). While the manipulator is moving, each section pumps out the water in the buoyancy chamber at a constant speed $v_{\mathrm{pump}}=0.8\;\mathrm{cm}/s$, providing buoyancy to help the manipulator lift the object. For comparison, perform the same movement on the same manipulator (but without BRS). The Reynolds number Re≈7100, so the drag coefficient Cd ≈1. The acting time of the movement is 10s and the step length of the solution is Δt=0.5 s (small enough to show the characteristics of tension). Solution results of the tension on each cable are shown in Figure 8, Figure 9, Figure 10, Figure 11 and Figure 12.

### 4.2. Discussion

Due to the large number of cables and the obvious difference in tensions’ time histories, it is difficult for us to obtain an intuitive feature that is generally valid for all curves from Figure 8, Figure 9, Figure 10, Figure 11 and Figure 12. Nevertheless, by comparing the two cases with and without BRS, we can see that the tensions on most cables are reduced by BRS. On several cables, the effect of BRS on reducing tension is very significant (for example, the average tension on cable 3—1 is reduced by 91.62%). We believe that is because the buoyancy balances the weight of the UCDM, which in general makes the motion require smaller driving forces. On a few cables, BRS increases the tension slightly (for example, the average tension on cable 5—2 increased by 18.62%). This is because these cables are located in the lower half of each section. When the buoyancy of a section is greater than the gravity of this sections, the cables in the lower half are pulled by the upward net buoyancy, hence the tensions are increased.

For underwater manipulators, the power required to perform actions is worthy of attention, because the power and energy that underwater robots can provide are relatively limited. During the motion of the UCDM, the sum of the power on all cables in a transient can be calculated by (23):

(23) $$P=\sum_{i=1}^{n}\sum_{j=1}^{3}T_{i,j}*\frac{\Delta L_{i,j}}{\Delta t}.$$

**Figure 13:**
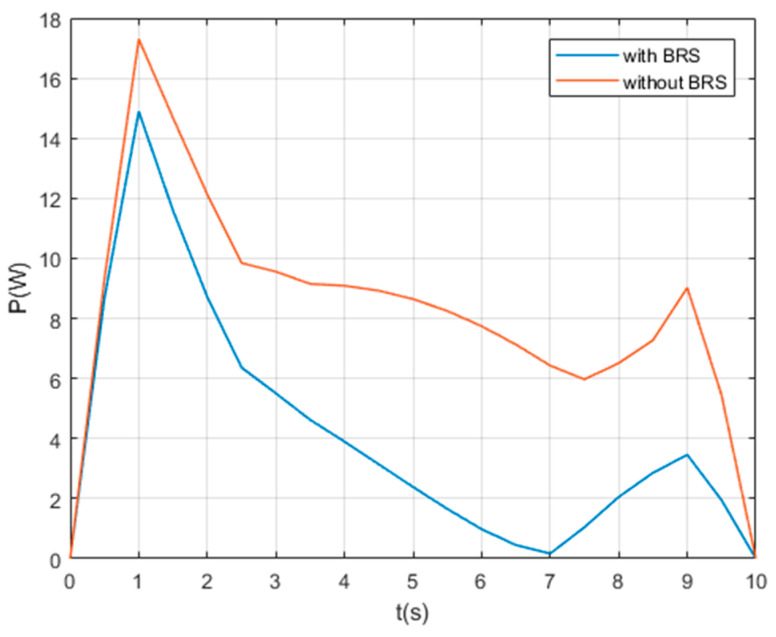
The time histories of the power of all cables.

This formula sums the effects of BRS on all cables and presents them on two curves, as shown in Figure 13. It is obvious that BRS significantly reduces the power consumption on cables. The average power is reduced by 51.04%, and the maximum power is reduced by 13.92%.

At t = 1 s, both cases are at the maximum power. This is within our expectations, because at this moment the manipulator has just begun to move. It has a large acceleration, so the driving forces have to overcome a large inertial force and water resistance. Similarly, at t = 9 s, each cable pulls the manipulator to stop the movement, so the power curve also has a local maximum here. Of particular concern is that at t = 7 s, the power with BRS drops to a minimum. We believe that these are because the net buoyancy of each section is close to 0 at this time, so each cable tension is near its minimum value (as shown in Figure 8). This result shows that if the buoyancy can be adjusted to dynamically match the load so that the net buoyancy of the manipulator is controlled close to 0, the driving force of each cable can be minimized. Using buoyant materials or air-filled chambers cannot accomplish this goal because their buoyancy is not adjustable. This result can be used to significantly reduce the tension of each cable, which is expected to greatly reduce the power of driving forces.

## 5. Conclusions

This article proposes an underwater cable-driven manipulator with a fluid-power buoyancy regulation system for underwater operations, and conducts dynamic analysis. Considering the buoyancy effect on the UCDM’s motion among all hydrodynamic factors, we analyzed the coupling relationship between cable tension, inter-joint force, buoyancy, and water resistance, established dynamic equations using Newton–Euler method, and numerically solved the time histories of the tension on each cable in a vertical movement. The results show that under the effect of BRS, the sum of the driving forces required to perform the movement is reduced by about 50%. Furthermore, by dynamically matching the load with buoyancy, the sum of the driving forces is expected to be reduced by up to 90%. This result indicates that BRS can reduce the energy demand of the UCDM, because the effect of buoyancy effectively balances the negative impact of the manipulator’s self-weight.

With BRS, it is expected to greatly increase the number of sections of the UCDM, which will enlarge its arm span and buoyancy regulation range. This improvement is feasible, but too many pump sets will make the system complex and hard to control. In general, we proposed an UCDM with high DOFs and low energy demand. This can be used in performing complex underwater actions and underwater tasks with limited energy supply. In future, the control strategy of distributed buoyancy regulation will be studied to optimize the power of the driving motors.

## Figures and Tables

**Table 1:** The specifications of the manipulator.

Characteristics	Value
Size (L × W × H)	1012 mm × 116 mm × 116 mm
Weight (moving parts)	2.55 kg
Maximum payload	4.5 kg
Load-to-weight ratio ^1^	1.76
Joint rotation range	±30°

^1^ The load-to-weight ratio is the ratio of payload to the weight of moving parts.
